# High Pressure Quenched Glasses: unique structures and properties

**DOI:** 10.1038/s41598-020-66418-7

**Published:** 2020-06-11

**Authors:** W. Dmowski, G. H. Yoo, S. Gierlotka, H. Wang, Y. Yokoyama, E. S. Park, S. Stelmakh, T. Egami

**Affiliations:** 1 Department of Materials Science and Engineering, University of Tennessee, Knoxville, TN 37996 USA; 2 Department of Materials Science and Engineering, Seoul National University, Seoul, 08826 Republic of Korea; 3 Institute of High Pressure Physics, Polish Academy of Science, Warsaw, Poland; 4 Materials Research Institute, Tohoku University, Sendai, Japan; 5 Materials Science and Technology Division, Oak Ridge National Laboratory, Oak Ridge, TN 37831 USA

**Keywords:** Engineering, Materials science, Physics

## Abstract

Zr-based metallic glasses are prepared by quenching supercooled liquid under pressure. These glasses are stable in ambient conditions after decompression. The High Pressure Quenched glasses have a distinct structure and properties. The pair distribution function shows redistribution of the Zr-Zr interatomic distances and their shift towards smaller values. These glasses exhibit higher density, hardness, elastic modulus, and yield stress. Upon heating at ambient pressure, they show volume expansion and distinct relaxation behavior, reaching an equilibrated state above the glass transition. These experimental results are consistent with an idea of pressure-induced low to high density liquid transition in the supercooled melt.

## Introduction

In crystals, temperature or pressure can promote a polymorphic structural transition from one phase to other, e.g. α-β in crystalline Zr. Similar transitions were observed in liquids and glasses, and they were named “polyamorphic” transitions^1,2^. For example, a first-order liquid-liquid phase change was reported in liquid phosphorous under pressure^3^. In metallic glasses, “polyamorphic” transition caused by pressure was reported in the Ce_55_Al_45_ alloy at room temperature (RT)^4^. A similar transition also attributed to *f*-electron delocalization^5^ (as in the *γ-α* transition in crystalline Ce) was observed in the multicomponent Ce_70_Al_10_Ni_10_Cu_10_ glass. However, since the electronic effect is stabilized by pressure, the structural transition is reversed when pressure is released, and the glasses regain the original structure.

**Figure 1:**
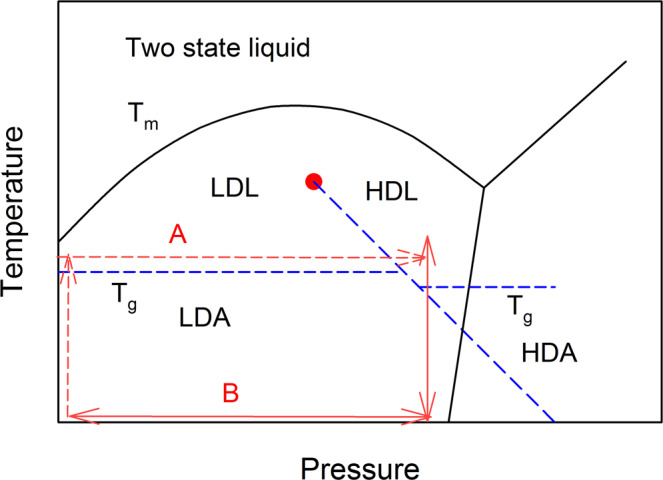
Idealized two-state liquid P-T phase diagram. LDL/HDL and LDA/HAD correspond to low/high density liquid/amorphous states and the dashed blue lines describe their hypothetical p-T range. Paths A and B are possible routes to reach HDL. Arrows indicate path direction with increasing or decreasing temperature or pressure. Path B was used to reach HDL in the super-cooled region. Sample was compressed at RT to the target pressure, heated up to the target temperature, cooled down under pressure to RT and then decompressed.

Recently we quenched supercooled metallic liquids under pressure to the glassy state and studied their structure by high-energy x-ray diffraction^6^. We showed that the High Pressure Quenched (HPQ) glasses represented the frozen state of the high-density supercooled liquid, by demonstrating that its structure was distinct from that of a glass quenched in ambient pressure. The HPQ glasses are stable at room temperature (RT) after decompression. The idea of the HPQ glasses is outlined schematically in the P-T phase diagram in the Fig. 1. The solid lines represent the idealized phase diagram of a two-state liquid^2^. In some systems the melting temperature (*T*_*m*_), after initial increase, reaches its maximum and starts to decrease with pressure. The pressure derivative of *T*_*m*_ is proportional to the ratio of the change in volume over the change in entropy (ΔV/ΔS). Therefore, a negative slope indicates that the volume decreases upon melting. This phenomenon is explained by a two-state liquid model in which there are fluctuating local states of low- and high-density liquid (LDL, HDL). With increasing pressure, the fraction of high-density liquid state becomes larger, resulting in the change in the slope from positive to negative. If crystallization is bypassed during cooling, a critical point is observed (a red dot in Fig. 1). The supercooled liquid develops a double minimum in its energy of mixing between LDL and HDL components, triggering separation into liquid polymorphs which are separated by a first-order transition line^2^. Further quenching results in glassy polymorphs corresponding to low- and high-density amorphous (LDA and HDA) states. It is worth noting that density driven L-L transitions are found often in the metastable supercooled liquid (SCL) range, well below the melting temperature^7^. The experimental difficulty lies in how to reach the metastable HDL and produce HD amorphous polymorph bypassing crystallization. Figure 1 illustrates two possible pathways. The first one (marked as A - red dashed line) is to heat a sample to a temperature within a SCL, increase pressure, quench to room temperature (RT) and decompress. However, this is technically challenging because, as we found before, some minimum pressure is needed to inhibit crystallization. It may be that LDL is more prone than HDL to crystallization under pressure, or simply some pressure is needed to suppress diffusion. The second path which we followed (marked as B in Fig. 1- red solid line) is to apply pressure at RT first and then raise temperature to reach supercooled HDL, then quench to RT and decompress. It seems that HDL is rather stable under pressure and this route successfully produces HPQ glasses. In this study we examine additional compositions of Zr-based glasses, report several physical properties of the HPQ glasses, and demonstrate that they are consistent with an idea of pressure-induced low density to high density liquid-to-liquid transition in the supercooled melt, as has been argued before^6^. Recent publication^8^ confirms our previous results on HPQ glass^6^.

## Results and Discussion

### Structure and pair distribution functions

**Figure 2:**
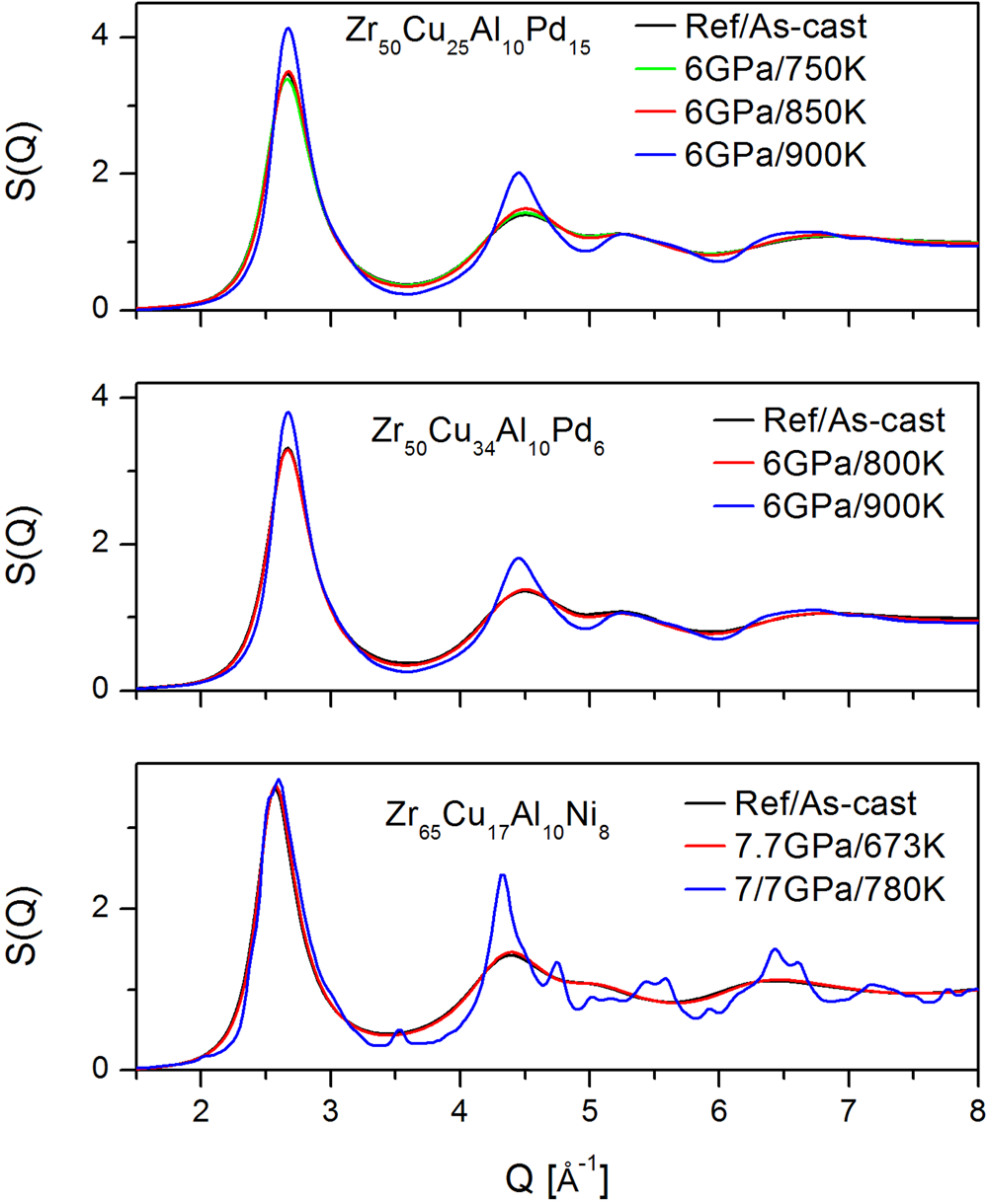
Structure function of selected glasses. The blue color shows sample that is partially or fully nano-crystallized.

**Figure 3:**
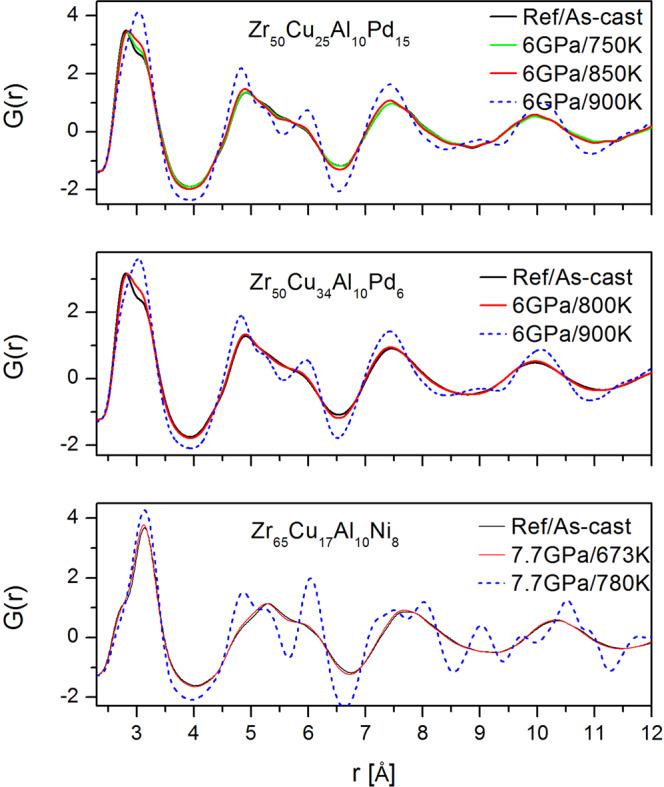
Reduced pair distribution function corresponding to S(Q) samples shown in Fig. 2. The dashed blue line indicates samples that are partially or fully crystallized.

We examined three Zr-based metallic glasses: Zr_50_Cu_50-x_Al_10_Pd_x_ (x = 6, 15) and Zr_65_Cu_17_Ni_8_Al_10_ quenched under pressure from a high temperature to RT. After decompression at RT, the HPQ samples were examined at ambient conditions using high-energy X-ray diffraction in transmission geometry to characterize the resulting state. Figure 2 shows example structure functions for Pd = 15, Pd = 6, and Zr65 after quenching under pressure from different temperatures. Zr65 samples are already crystallized under pressure at ~820 K. Pd = 15, 6 samples remain glassy up to 850 K/6 GPa. The *S*(*Q*) of the 900 K/6 GPa sample is questionable due to the prominent height of the peaks. Indeed, TEM examination revealed that it is partially nano-crystallized. The structure functions in Fig. 2 show small changes in the glassy state, mainly sharpening in peaks and valleys and small phase shifts. The partially or fully crystallized samples exhibit distinct, sharp features in *S*(*Q*). Complex peak structure is clearly seen for crystallized Zr65 sample. Combined information from *S*(*Q*), PDFs (Fig. 3) and HRTEM (for Pd = 15 6 GPa/T = 900 K) were used to establish experimental conditions under which high pressure quenched glasses (HPQ) can be prepared.

**Figure 4:**
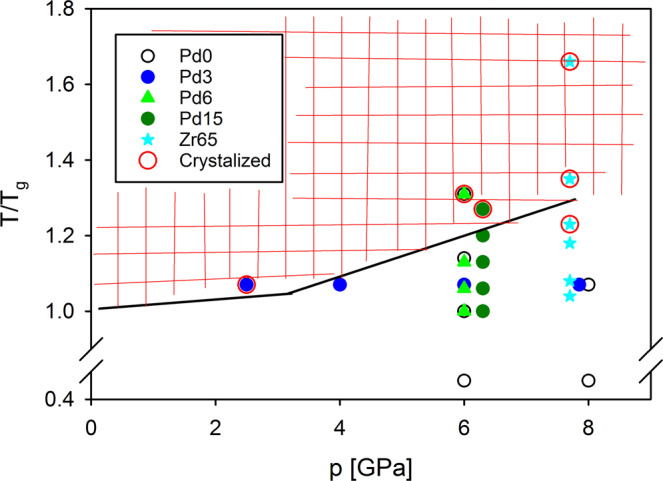
Experimental conditions used in the high-pressure quenching process. Temperature is normalized to Tg. Samples are Zr50Cu40-xPdxAl10 and Zr65Cu17Ni8Al10. Red circles around data point indicate that resulting samples was partially or fully crystallized.

Figure 4 illustrates the range of pressure and temperature applied in our experiment and the resulting sample state after quenching from high temperature under pressure. The presented data are combined with previous results for x = 0 and x = 3 samples^6^. Different symbols correspond to different compositions as indicated in the legend. The red circle around the symbol signifies that the sample is partially or fully crystallized. As was shown before^6^, application of some minimum pressure is needed to suppress crystallization in the supercooled liquid, ~3 GPa, and HPQ samples are glassy if higher pressure is applied while heating to SCL. It appears that low density SCL (see Fig. 1) is prone to crystallization under pressure; however, this point requires further studies. For the Zr65 sample, we also attempted to quench under pressure from above the liqidus temperature (>1160 K); however, all samples turned out to be crystallized and are not shown in Fig. 4. This could be because solidus is higher than critical point in the schematic diagram in Fig. 1 and quenching from the super-cooled liquid may be essential^7^. More experimental studies, however, are necessary.

**Figure 5:**
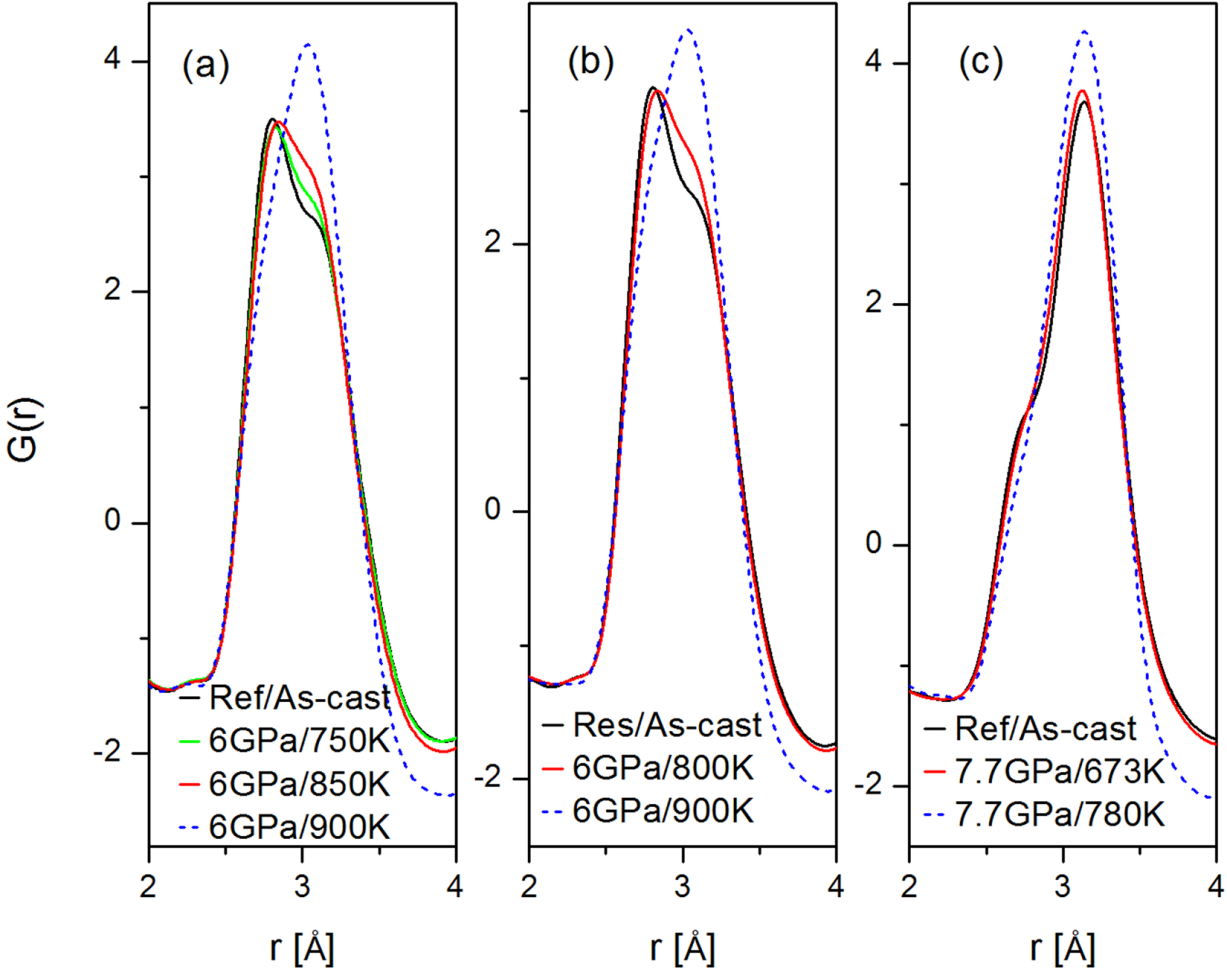
First PDF peak showing first shell atomic ordering change for HPQ glasses: (**a**) Zr50Cu25Pd15Al10, (**b**) Zr60Cu34Pd6Al10, (**c**) Zr65Cu17Ni8Al10. The blue dashed line is for partially or fully crystallized samples.

Figure 5 shows the first peak in the reduced pair distribution function *G*(*r*) for the HPQ and reference samples. The main contribution to the right shoulder of the *G*(*r*) fist peak comes from the Zr-Zr partial PDFs with the expected average distances at ~3.12 Å in the reference sample (Zr-Cu ~2.75 Å, Pd-Pd ~2.78 Å). The PDF of the HPQ samples differs from the reference (as-cast sample) after decompression at RT, indicating different structure. The new results are consistent with the previously published data for the x = 0 and x = 3 samples^6^ despite higher Pd or Zr content. The second subpeak of the first PDF peak becomes higher and shifts slightly towards smaller *r*. Thus, the change in the distribution of atomic distances is such that those in the right shoulder of the first peak in *G*(*r*) move towards the center after quenching under pressure. The right shoulder corresponds to Zr-Zr pairs and this shift indicates that the average distance and spread of Zr-Zr pairs become smaller. More details describing the shift of the first G(r) peak are shown in the Supplement figures S1, S2 and S3. In addition, all peaks of *G*(*r*) become narrower, and the amplitudes of oscillation become larger as seen in Fig. 3. Overall changes in the G(r) beyond first peak (so called medium-range-order) are small and difficult to uniquely quantify. For example, significant changes in MRO were observed in Pd-based glass during liquid-liquid transition^9^. We attributed structural changes in the PDF mainly due to Zr changing its packing environment under pressure. It is well known that Zr can form distinct phases under pressure, temperature, and strain e.g.^10,11^. As we discussed before^6^, Zr bonding has a covalent character and can slightly change its packing environment depending on pressure (e.g. alpha, omega or beta phase in crystalline Zr). Indeed, calculations showed that Zr in metallic glass has *d*-electron density^12^ around 3. Application of high pressure in the liquid state reduces covalency-induced distortions resulting in dense-packed amorphous structure with higher density, as corroborated by a density measurement. Thus, the distortions in Zr-Zr environment observed in regular glasses are reduced by pressure at high temperature. This structural change is similar to the one observed in silicon^13^, germanium^14^ and ice^15^, where pressure drives the low-density state with covalent bonding to a high-density state with better packed structure.

### Density and thermal studies

**Figure 6:**
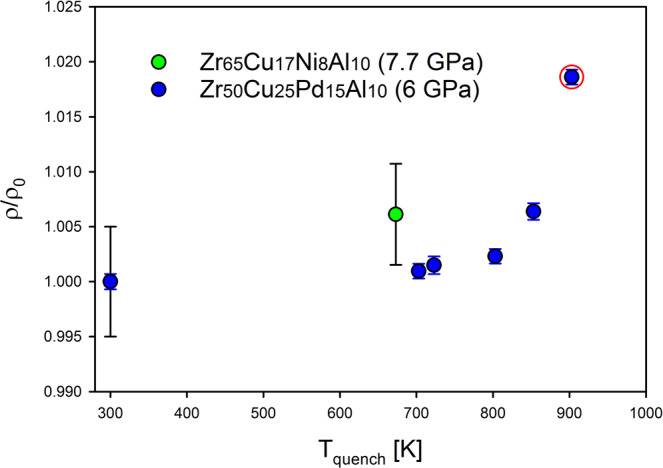
Normalized physical density change for several samples plotted against the temperature the sample was quenched from. The red circle denotes a sample that is partially nano-crystallized. Zr_65_Cu_17_Ni_8_Al_10_ was quenched under 7.7 GPa, whereas Zr_50_Cu_35_ Pd_15_Al_10_ was quenched under 6.0 GPa.

**Figure 7:**
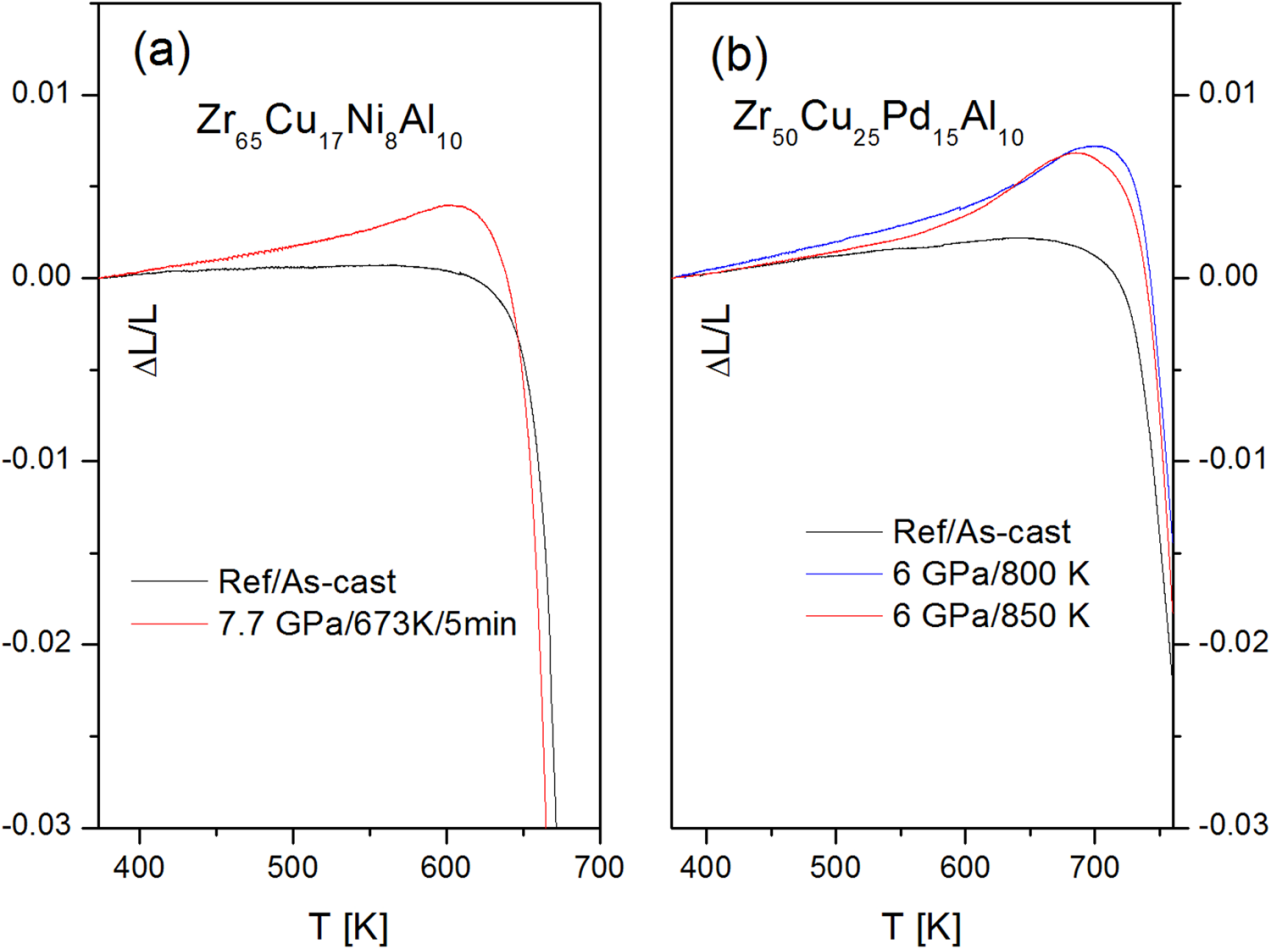
Normalized to RT change in sample dimension upon heating to the glass transition for (**a**) Zr65Cu17Ni8Al10 and (**b**) Zr50Cu25Pd15Al10 samples. Black lines represent as-cast (reference) state for each composition. Color lines correspond to quenching under pressure from a given temperature.

In Fig. 6 we show densities measured for the recovered HPQ glasses, normalized to that of the as-cast sample. It is seen that there is a clear tendency of increasing density for samples quenched under pressure. These results are consistent with the idea that HPQ glasses are quenched from the high-density liquid (HDL) and represent the high-density amorphous phase (HDA) as schematically described in Fig. 1. We showed before by structural studies (PDF) that annealing at *T*_*g*_ transforms HPQ glasses back into ambient pressure glasses^6^. This observation is also confirmed by the TMA studies. Figure 7 shows linear expansion normalized to room temperature value for the Zr65 and Pd = 15 samples in the as cast and HPQ states. It is observed that on approaching *T*_*g*,_ HPQ glasses increase their dimensions, expanding much more than as cast samples. Together with density data, this confirms the idea that hydrostatic pressure in the SCL region leads to a new state with higher density with better packing.

**Figure 8:**
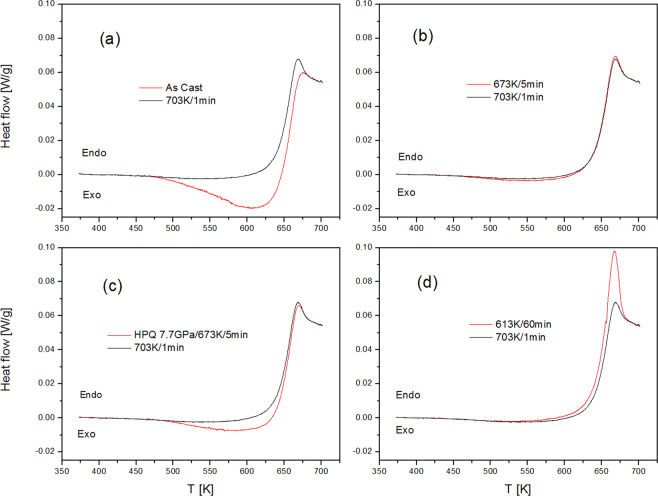
DSC traces for the Zr65Cu17Ni8Al10 glasses in different thermal state (**a**) as-cast, (**b**) as-cast sample equilibrated for 5 mins at 673 K, (**c**) HPQ glass quenched from 673 K under 7.7 GPa pressure, (**d**) as-cast sample annealed at 613 K for 60 mins. The red line is an initial measurement for each sample by heating with 20 K/min to 703 K. The black line denotes reference state obtained by subsequent cooling from 703 K with 50 K/min.

Another distinct feature of HPQ glasses is revealed by calorimetry measurements. Figure 8 shows the heat evolution for four Zr65 samples: as-cast, annealed at 670 K (5 mins), 610 K (60 mins) and HPQ glass (670 K/7.7 GPa/5 min). Each scan is plotted together with an equilibrated reference state (at 703 K/1 min). The difference between the red and black curves illustrates the relaxation spectra for each sample. The as-cast sample (Fig. 8a) exhibits typical behavior for a rapidly quenched metallic glass^16^. Below *T*_*g*_ (638 K) there is a significant exothermal heat evolution reflecting a well-known process of sub-*T*_*g*_ structural relaxation. Once the sample reaches super-cooled region, the endothermic process starts because glass has to attain equilibrium volume of the liquid in this temperature rangee.g.^17^. Thus, above *T* ~ > 680 K all samples achieve the same thermodynamic state at this heating rate. Figure 8b) shows the data for a sample quenched from 673 K, which has similar thermal history as the HPQ glass. This sample exhibits small exo- and endothermal heat flows in the spectrum but is very close to the reference equilibrium state. The sample in the relaxed state (Fig. 8d) that underwent sub-*T*_*g*_ annealing takes heat from the environment to increase its internal energy over the whole temperature range before reaching the equilibrated state in the SCL. The relaxation spectrum (the difference between the red and black curves) of the HPQ glass (Fig. 8c) has a significant exothermal component as compared to the ambient pressure sample with similar thermal history (Fig. 8b). It is qualitatively similar to the as-cast sample but with a smaller amplitude. On the other hand, the relaxation spectra of the HPQ and sub-*T*_*g*_ relaxed samples are very different. This result confirms our findings^6^ that the structure of the HPQ glass is distinct from the relaxed structure, despite both having better packing, and that the structural changes induced by pressure in the SCL are not caused by structural relaxation or crystallization. The relaxation spectra of metallic glass are almost exclusively attributed to the “free volume” annealing or creation upon heating to the SCL e.g.^13^. However, the results for HPQ glass clearly show that it is not the case. HPQ glass has higher density and, as seen from Fig. 7, it is expanding (increasing its volume) upon approach to *T*_*g*_, yet its relaxation spectrum shows a net exothermic effect. Volume expansion in glasses (i.e. below *T*_*g*_) is typically coupled with heat absorption, as seen in the Fig. 8(d); therefore, it is most likely that in the HPQ sample there are at least two opposite energetic processes, with a heat release being the larger. It can be expected that HPQ glass is in a higher energy state than ambient pressure glass since considerable energy has been injected into the system by volume compression and structural rearrangements under pressure. Upon heating at ambient pressure, as in DSC experiments, HPQ glass relaxes eventually to the equilibrium, ambient pressure state. The exothermic component could be just a release of energy gained by a reverse transition to the LDA.

### Mechanical properties

**Figure 9:**
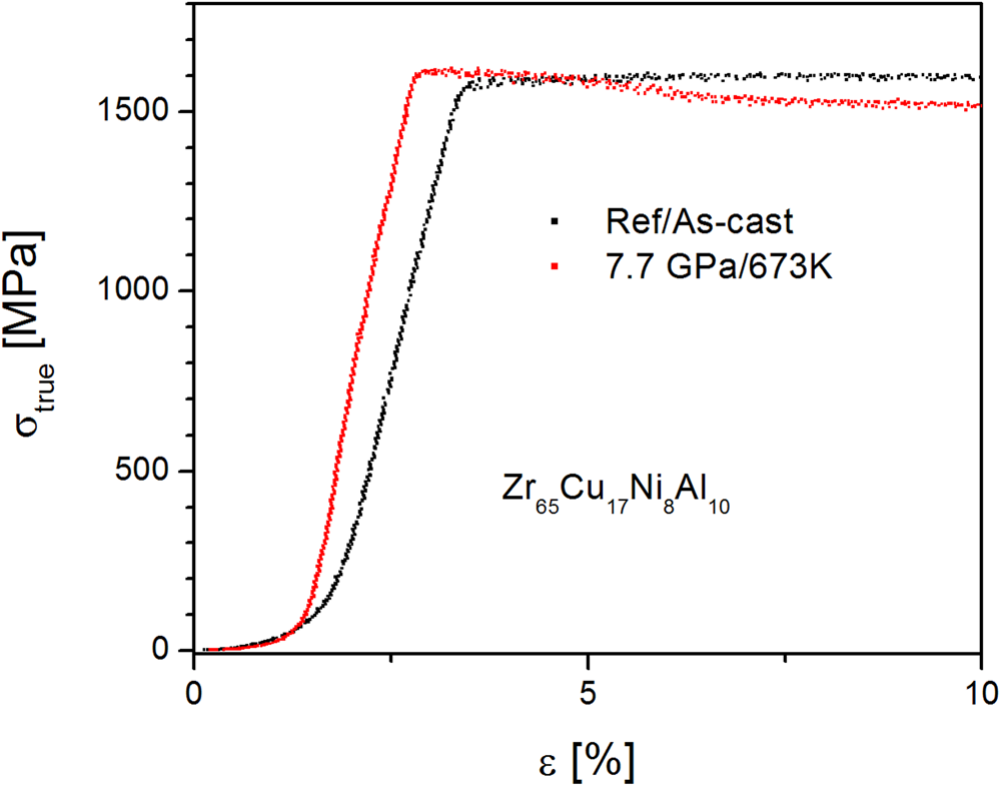
Stress-strain curves for Zr65Cu17Ni8Al10 sample. The HPQ glass exhibits larger yield stress and larger Young’s modulus.

The mechanical properties of HPQ glasses differ from those of the ones quenched at ambient pressure. As is demonstrated in the Table 1, hardness is slightly increased similarly to the sub-*T*_*g*_ relaxed glasses, reflecting better packing and increased density. Interestingly, we also found that yield strength and Young’s modulus are higher for the HPQ Zr65 sample. Figure 9 shows the compression curve of the Zr65 glass in the as-cast and HPQ states. It is seen that HPQ glass shows steeper slope (higher Young’s modulus) and yield strength goes from 1562 to 1605 MPa, or by ~ 2.8%. Young’s modulus is changed from 95 to 98.5 GPa as determined by nanoindentation. Similar results (higher yield strength and Young’s modulus) were recently observed^8^ for HPQ glass Zr_50_Cu_40_Al_10_.

**Table 1:** Hardness of the HPQ samples.

	Zr65 AC	Zr65 673 K/7.7GPA	Zr65 703 K/7.7 GPa	Pd15 AC	Pd15 803 K/6 GPa	Pd15 853 K/6 GPa
H [GPa]	5.38 ± 0.08	5.40 ± 0.13	6.05 ± 0.07	7.08 ± 0.15	7.23 ± 0.37	7.29 ± 0.59
H [Hv]	503 ± 7	525 ± 9	554 ± 7	623 ± 10	653 ± 6	674 ± 7

### Partially crystallized sample

**Figure 10:**
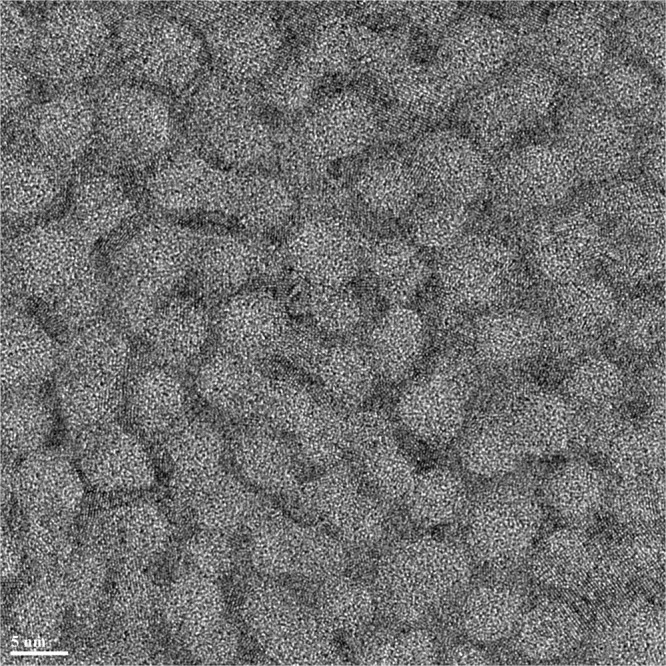
HRTEM micrograph showing structure of Zr50Cu25Al10Pd15 HPQ sample quenched from 900 K. The length scale is 5 nm. The microstructure exhibits clear lattice fringes in the boundaries between larger domains/grains with glassy structure. Such microstructure results in structure function shown in Fig. 2 with blue line, with enhanced S(Q) peaks.

As we noted before, the structure function of Pd = 15 glass at *T* = 900 K and *P* = 6 GPa (blue line Fig. 2) looks peculiar. It is similar to the glassy one but has higher peaks; in particular, the enhanced second peak intensity suggests that some nano-crystallization might have occurred. This assessment is confirmed by the PDF shown in Fig. 3. It is frequently difficult to discriminate partially nano-crystallized samples from a pristine glass using only the diffraction data, especially if grain size is in nanometer range. We examined this sample using HRTEM. Figure 10 reveals an intriguing microstructure. The length scale on the micrograph corresponds to 5 nm. There are glassy islands or domains in the structure ~5–10 nm, separated by boundaries that are clearly crystalline, less than 1–3 nm thick. Typically, crystallization in glasses advances through nucleation and growth of a crystalline phase in the amorphous matrix. Here, crystallization under pressure results in precipitation of continuous network of nanocrystalline phase. This peculiar behavior may be related to the Pd content in this glass. It was observed previously that nano-crystalline precipitates are likely to occur in Zr-based glasses that contain 10 at% of Pd^18^. It is also possible that supercooled liquid decomposes, resulting in selected crystallization, as observed in multicomponent Zr rich glasses e.g.^19,20^; however, it is rather difficult to separate decomposition from crystallization^21^ and crystallization itself depends strongly on kinetics and may change nucleation and growth modes depending on annealing temperature and/or pressure, e.g^22^.

### HPQ glasses

The HPQ glasses have a different structure and mirror the pressure-induced structure in the supercooled liquid state. The structural features are consistent with those previously reported for HPQ Zr-based glasses. The increase in physical density, volume expansion upon heating, and the distinct heat evolution confirms our idea that the HPQ glasses are high density polymorphs of the ambient pressure glasses. These glasses exhibit higher stiffness and hardness, and the compression test on Zr65 shows an increase in yield strength and Young’s modulus, which could be a common feature of HPQ glasses

## Methods

### Samples

In this work we examined three Zr-based metallic glasses: Zr_50_Cu_50-x_Al_10_Pd_x_ (x = 6, 15) and Zr_65_Cu_17_Ni_8_Al_10_. The metallic glass samples were prepared by a tilt-casting method as described in Ref. ^23^ The glass transition temperature (*T*_*g*_) and crystallization temperature (*T*_*x*_) for Pd-containing glasses are very close to each other (*T*_*g*_ = 708 K and *T*_*x*_ = 723 K at heating rate of 20 K/min for both x = 6 and 15), whereas the Zr65 glass has a relatively large supercooled region with *T*_*g*_ = 638 K and *T*_*x*_ = 753 K at heating rate 20 K/min. Glassy structure was verified by a high energy X-ray diffraction in transmission geometry and thermal (DSC), and dilatometry (TMA) measurements.

### High pressure quenching

Cylindrical shaped samples with a height ~<4 mm and ~3 mm diameter were used in the high pressure quenching experiments as described before^6^. The HPQ setup is described in Ref. ^24^ The sample was inserted into a graphite tube and padded with boron nitride as filler. The calibration of pressure was carried out using standard compounds and the temperature was monitored using thermocouple. The temperature has uncertainty of ±25 K. Each sample was compressed at RT to the final pressure and heated to reach the target temperature, held for 3–5 min to equilibrate, and then cooled down to RT. At room temperature, the samples were decompressed and removed from the compression cell. The approximate cooling rate in the cell is ~20–50 K/min depending on the temperature range, i.e. higher at high T and lower below ~470 K.

### Diffraction and structural data analysis

High-energy X-ray diffraction experiments were performed at the beamline 6-ID of the Advanced Photon Source, Argonne National Laboratory. The experimental setup and data processing are similar to previously described^6,25^. The incident beam energy was tuned to 100 keV, the beam size was 0.2 × 0.2 mm^2^, and a 2D stationary detector was placed ~40 cm behind the sample. The samples had comparable thicknesses, (0.5–0.6 mm) ensuing negligible systematic errors due to background and absorption corrections. The collected data had been normalized to incident beam monitor recorded by an ion chamber in front of the sample. Azimuthally integrated intensities were processed using the pdfgetX2 software^26^ to obtain the structure function, *S*(*Q*), up to Q ~ 23 Ǻ^−1^,where *Q* = 4*πsinθ/λ*, *θ* is the diffraction angle and *λ* is the x-ray wavelength. The background, multiple scattering and Compton scattering were removed, and data had been normalized to the absolute electron units. Fourier transformation of *S*(*Q*) produced the reduced pair distribution function, *G*(*r*), by

1 $$G(r)=4\pi r(\rho (r)-{\rho }_{0})=\frac{2}{\pi }\int Q[S(Q)-1]\,\sin (Qr)dQ$$

where ρ(*r*) is the pair density function (PDF) and *ρ*_0_ is the atomic number density.

### Density and thermal analysis

The physical density of the sample was measured using the Archimedes method (Mettler Toledo micro-balance) and as described in Ref. ^27^ Since sample weight is small, the absolute density value is prone to inaccuracy; however, relative values have smaller errors. Thermomechanical Analyzer by TA Instruments, model TMA Q 400, was used to examine thermal expansion of the sample. Study was performed in compression mode with a 1 N load on cantilever at a 5 K/min heating rate. Thermal analysis was done using Perkin-Elmer DSC 7. Because the SCL range is very limited for Pd containing samples, DSC studies were carried out on Zr65 glass. Initially, the Zr_65_Cu_17_Ni_8_Al_10_ sample was heated to establish characteristic temperatures such as *T*_*g*,_ (~638 K), the range of the supercooled region, and the onset of crystallization *T*_*x*_ (753 K). The temperature of 703 K, in the supercooled region, was chosen as a reference state. Two as-cast samples were annealed in DSC: for 60 min (at 613 K) and 5 min (at 673 K). DSC scans were performed on four samples: as-cast, HPQ, and the two annealed. Each sample was heated to 703 K (at 20 K/min), kept for 1 min, and cooled down with 50 deg/min cooling rate. Relaxation time at 703 K (~65 K above the glass transition) is short and thus every sample became equilibrated to the same reference state^28^. After cooling down, each sample was heated above the crystallization temperature, cooled down and the crystallized sample was scanned to establish baseline for each measurement.

### Mechanical properties and imaging

Hardness and nanoindentation measurements were carried out using Emco-Test (Dura Scan 70) and Hysitron Triboindenter (TI 950), respectively. Compression tests were done on 2.5 mm samples with 2 mm^2^ cross-section using a custom-built load frame. HR-TEM (JEM-2100F) was used to image the partially crystallized sample.

## Supplementary information

Supplementary Information.

## Data Availability

The data is available in form of Excel files from wdmowski@utk.edu on e-mail request.
