# Supplementary material for: High Pressure Quenched Glasses: unique structures and properties
Source: Sci Rep. 2020 Jun 11;10:9497. doi: 10.1038/s41598-020-66418-7 (PMC7289830; doi:10.1038/s41598-020-66418-7)
Supplement: Supplementary file 1 — Supplementary Information. [file 41598_2020_66418_MOESM1_ESM.pdf]

Supplemental Information to "High Pressure Quenched Glasses: unique structures and properties."

W. Dmowski, G. H. Yoo, S. Gierlotka, H. Wang, Y. Yokoyama, E.S. Park, S. Stelmakh and T. Egami

1. Shift of the PDF peaks caused by quenching under pressure

To illustrate the shift of the first atomic we plot $T(r) = 4 \cdot \pi \cdot r \cdot \rho(r) = G(r) + 4 \cdot \pi \cdot r \cdot \rho_0$, where ρ_0 is number density of atoms and G(r) is a reduced pair density function. The function T(r), describing distribution of atoms for isotropic sloid with spherical atoms should have Gaussian form [e.g. 1]. Figures S1, S2 and S3 plot *T(r)* for a reference sample, the HPQ sample and the difference between HPQ and Ref multiplied by 10. The green vertical line corresponds the peak position corresponding to the Zr-Zr side of the distribution function ~3.13 Å. The dash black line is the difference. It can be seen that to the right side of the vertical line the difference is mainly negative. It clearly means that the Zr-Zr side of the peak moves towards smaller *r* values shifting position and reducing spread of the Zr-Zr distribution. The difference is qualitatively similar for all three glasses; however, the details are dependent on the chemical composition. The redistribution of Zr-Zr atoms will likely affect Cu-Zr chemical ordering and resulting partial PDFs. The center peak in the difference is the result of changed overlap of Zr-Zr and Zr-Cu partial distribution functions. Qualitatively this was illustrated by a result of molecular dynamic simulations as shown in Supplement of ref. 2 (Fig. S3).

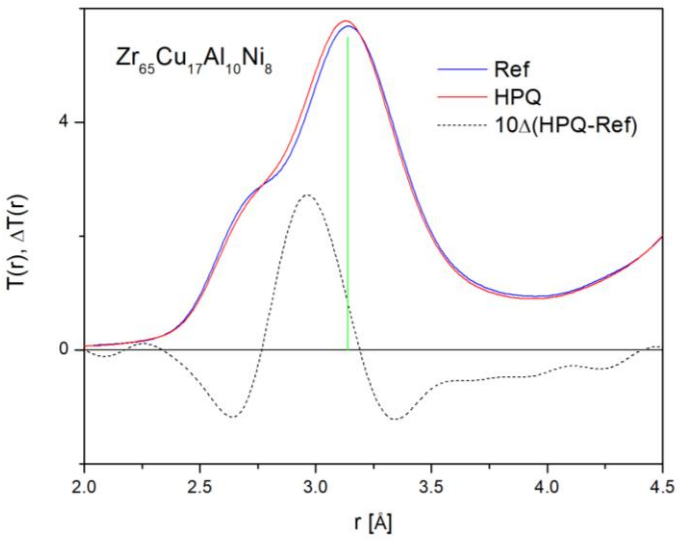

Fig. S1. Shift of the first atomic shell after HPQ process (T=673K,7.7 GPa) illustrated by difference in T(r) for $Zr_{65}Cu_{17}Al_{10}Ni_8$.

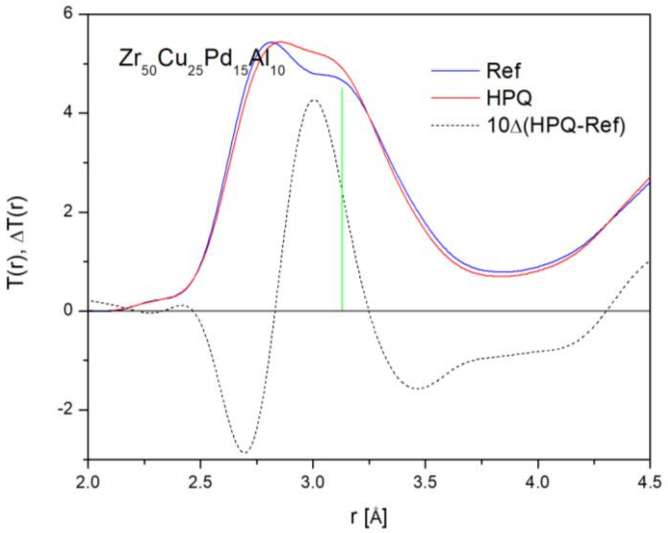

Figure S2. Shift of the first atomic shell after HPQ process (T=850K, 6 GPa) illustrated by difference in T(r) for $Zr_{50}Cu_{25}Pd_{15}Al_{10}$.

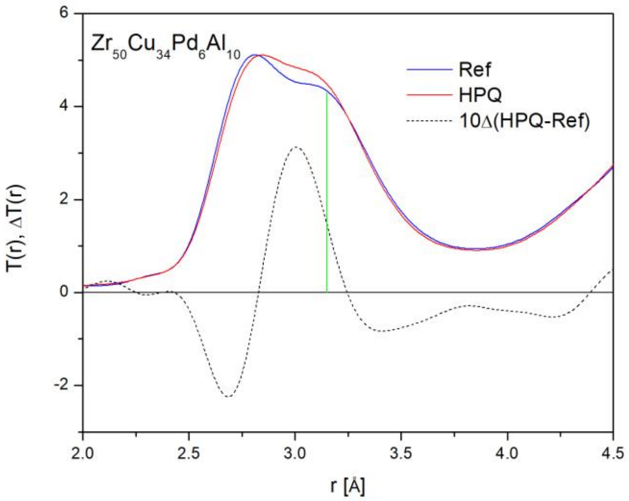

Figure S3. Shift of the first atomic shell after HPQ process (T=723K, 7.7 GPa) illustrated by difference in T(r) for $Zr_{50}Cu_{34}Pd_{6}Al_{10}$.

Supplement References

1. Ma, D. et al. Nearest-neighbor coordination and chemical ordering in multicomponent bulk metallic glasses, *Appl. Phys. Lett.* ***90***, 211908 (2007).

2. Dmowski, W. et al. Pressure Induced Liquid-to-Liquid Transition in Zr-based Supercooled Melts and Pressure Quenched Glasses, *Scientific Reports* ***7***, 6564 (2017).
